# Supplementary material for: VSIG4 inhibits proinflammatory macrophage activation by reprogramming mitochondrial pyruvate metabolism
Source: Nat Commun. 2017 Nov 6;8:1322. doi: 10.1038/s41467-017-01327-4 (PMC5673889; doi:10.1038/s41467-017-01327-4)
Supplement: Supplementary file 1 — Supplementary information [file 41467_2017_1327_MOESM1_ESM.pdf]

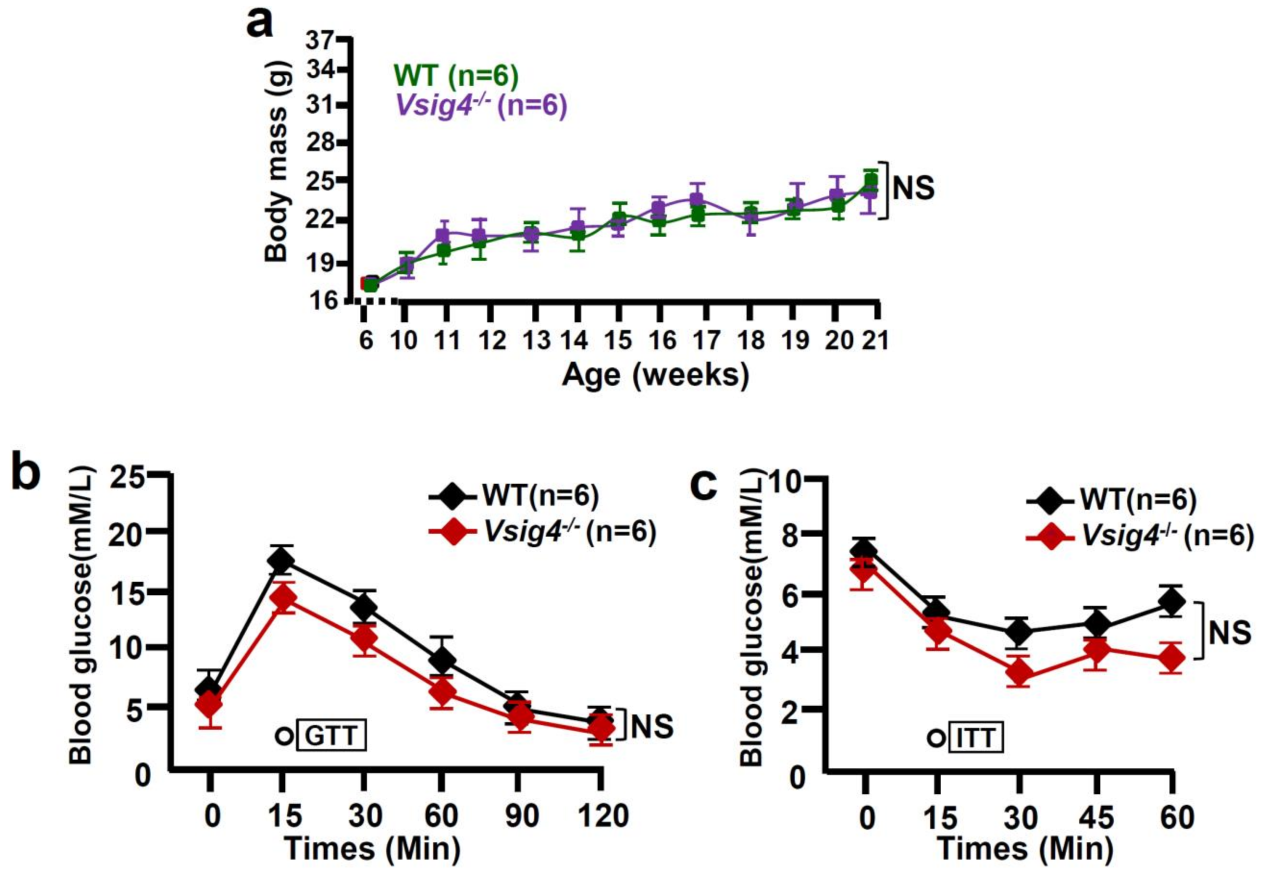

Supplementary Figure 1 *Vsig4* deficiency does not affect body mass and glucose levels under NCD-feeding condition. *Vsig4*$^{-/-}$ mice and their C57BL/6 WT littermates were fed with NCD, (**a**) body weight was measured during the entire course of NCD feeding, (**b**) GTT experiments, (**c**) ITT experiments. Error bar, s.e.m. NS, p>0.05 (Student's t-test). Data are representative of three independent experiments.

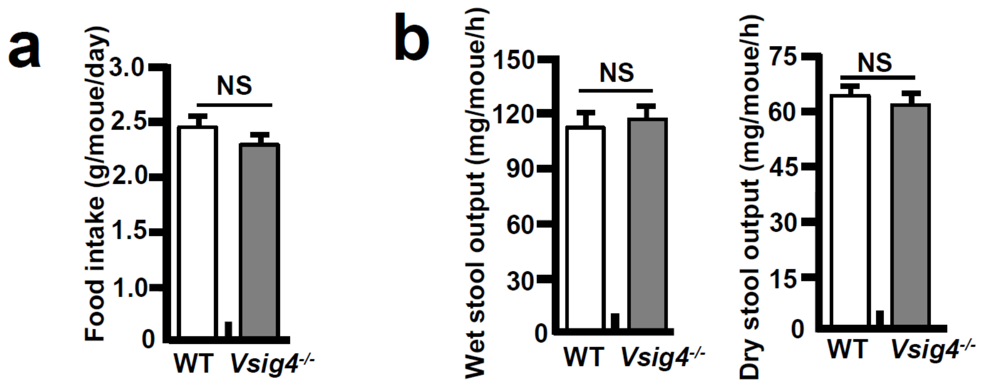

Supplementary Figure 2 *Vsig4* deficiency does not affect food intake and stool output in mice under HFD-feeding condition. *Vsig4*$^{-/-}$ mice and their C57BL/6 WT littermates were fed with HFD for ten weeks to induce obesity, (**a**) food intake, and (**b**) stool output was compared, n=6 *per* group. Error bar, s.e.m. NS, $p>0.05$ (Student's *t*-test). Data are representative of three independent experiments.

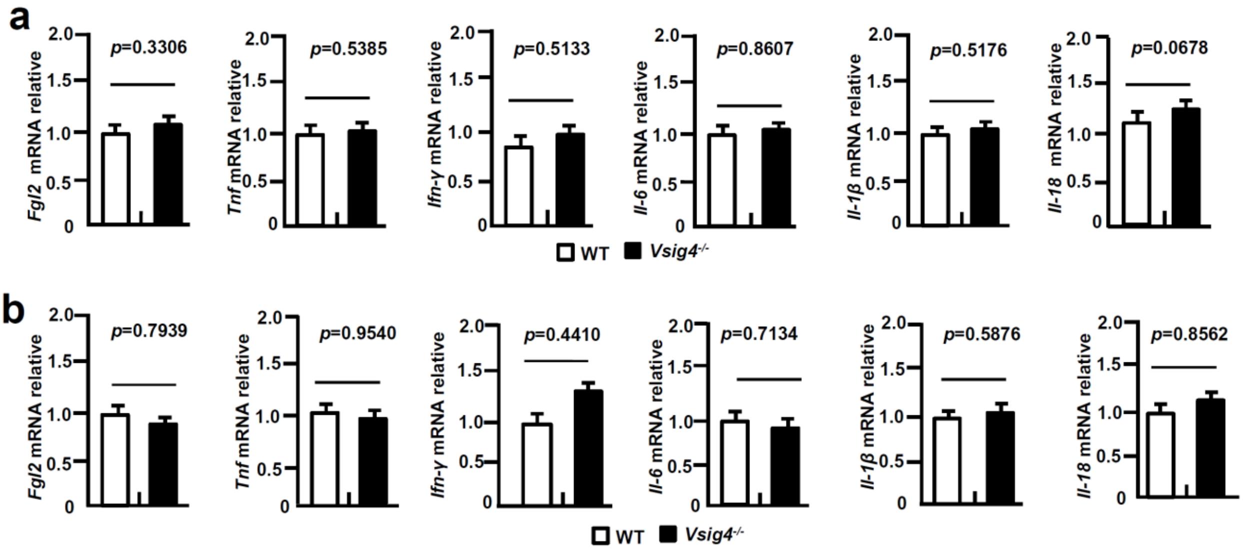

Supplementary Figure 3 *Vsig4* does not affect the transcription of proinflammatory mediators under normal condition. The transcription of proinflammatory cytokines in (**a**) PEMs, and (**b**) liver tissues from *Vsig4*$^{-/-}$ and WT mice under normal condition was detected by qRT-PCR, n=5 *per* group. Error bar, s.e.m. $p>0.05$ was not significant different (Student's *t*-test). Data are representative of three independent experiments.

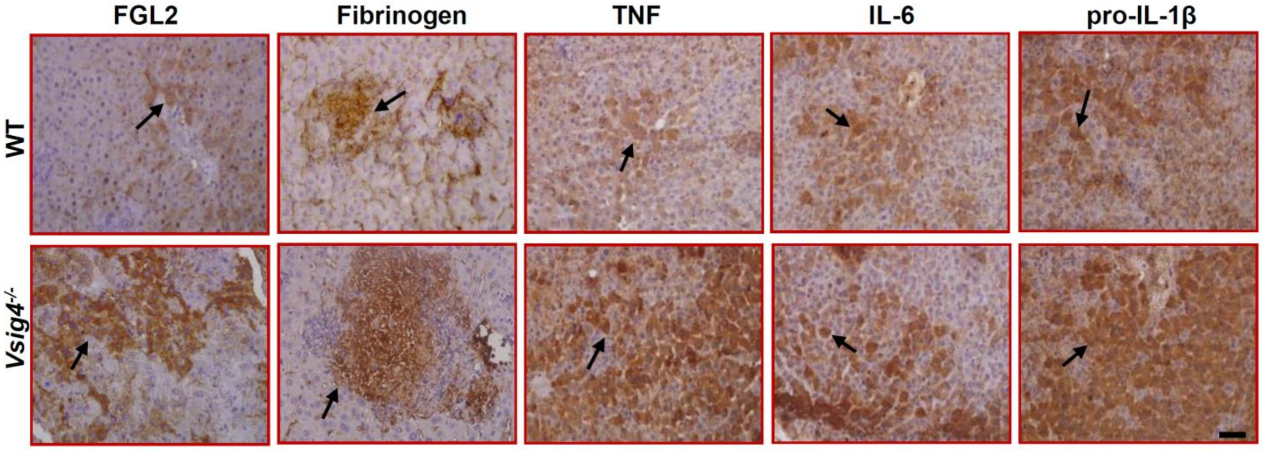

Supplementary Figure 4 Enhancing proinflammatory cytokine expression in *Vsig4*$^{-/-}$ livers at 48h of MHV-3 infection. The expression of FGL2, fibrinogen and proinflammatory factors in liver tissues was measured by immunohistochemistry at 48h of MHV-3 infection in mice, n=5 *per* group, scale bar=20μm. Arrow indicated positive cells. Data are representative of three independent experiments.

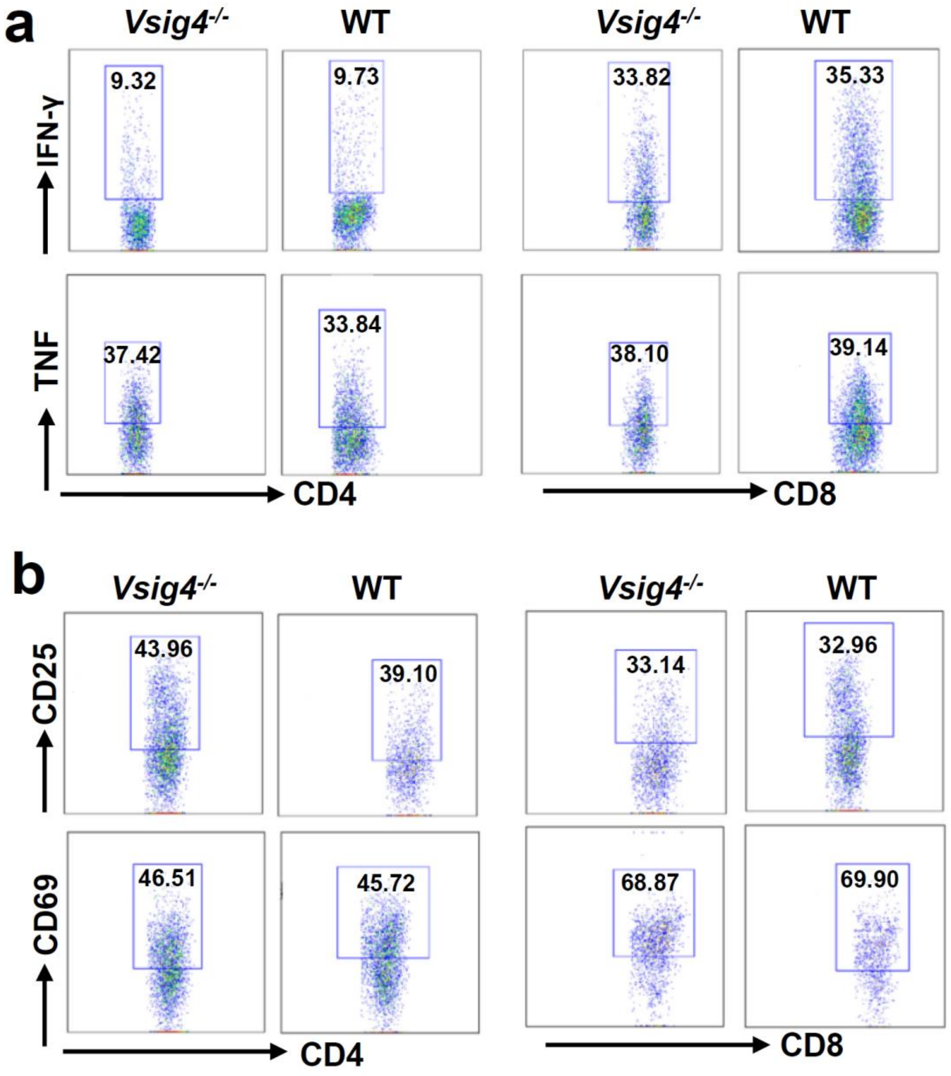

Supplementary Figure 5 The activation status of T cells was not affected by VSIG4 signalling. T cells were isolated from the liver tissues of MHV-3 infected *Vsig4*$^{-/-}$ and C57BL/6 WT mice at 72h, flow cytometry analyzed **(a)** the secretion of proinflammatory cytokines and **(b)** the expression of activation makers (CD25 and CD69) on CD4^{+} and CD8^{+} T cells . Data from one representative of five mice per group are shown. Data are representative of three independent experiments.

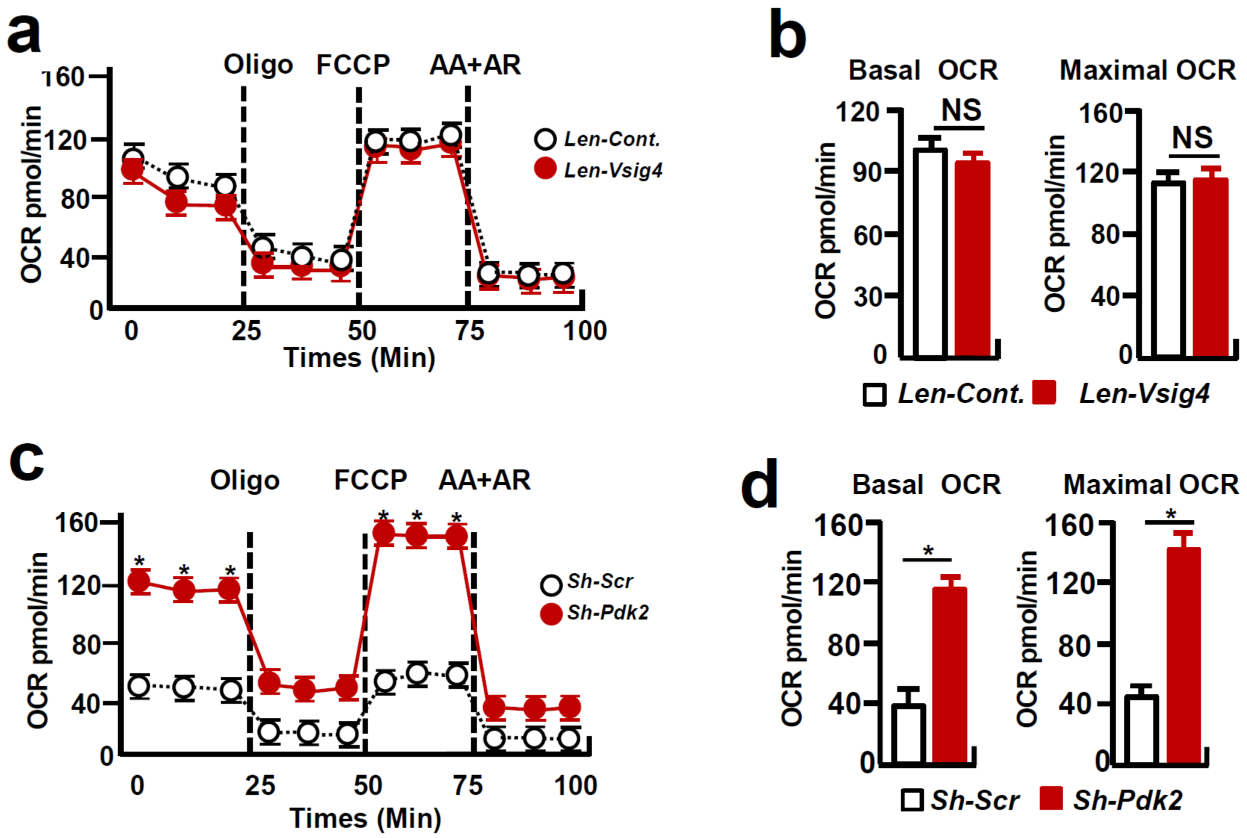

Supplementary Figure 6 Glucose oxidation in RAW264.7 cells under normal condition. RAW264.7 cells were infected with lentiviral control vectors (*Len-cont.*) or vectors encoding *Vsig4* (*Len-Vsig4*), cells were seeded at 2.0x10^4 cells/well in 8-well plates for 5h to allow adherence to the plate, (**a**) OCR in RAW264.7 cells of *Len-cont*.and *Len-Vsig4.* (**b**) Basal and maximal OCR of the indicated conditions was plotted in bar graphs. The expression *Pdk2* in RAW264.7 cells was silenced by shRNA, and (**c**) OCR was measured on Seahorse XFp. (**d**) Basal and maximal OCR of the indicated conditions was plotted in bar graphs. N=5 *per* group. Error bar, s.e.m. **p*<0.05 and NS, *p*>0.05. (Student's *t*-test). Data are representative of three independent experiments.

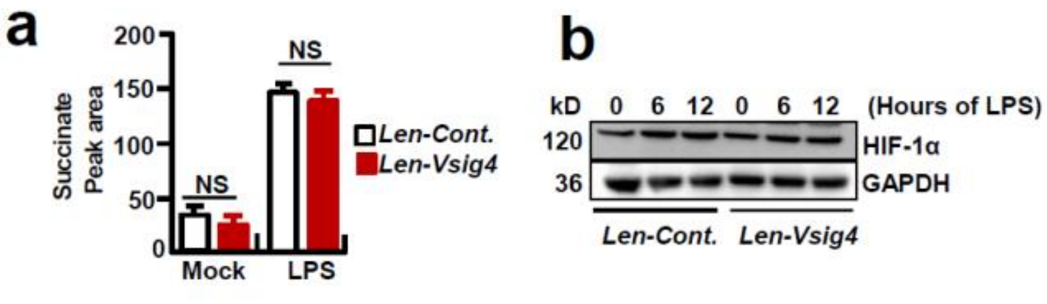

Supplementary Figure 7 Succinate accumulation and HIF-1α expression in macrophages was not affected by VSIG4 signalling. *Vsig4*$^{+}$Raw264.7 cells and their control counterparts were treated in the presence or absence of LPS (2μg/ml) for 6h, (**a**) the concentration of succinate was compared by Colorimetric/Fluorometric assay, n=5 *per* group. (**b**) Western-blot of HIF-1α protein in LPS treated cells. Error bar, s.e.m. NS, $p>0.05$ (Student's *t*-test). Data are representative of three independent experiments.

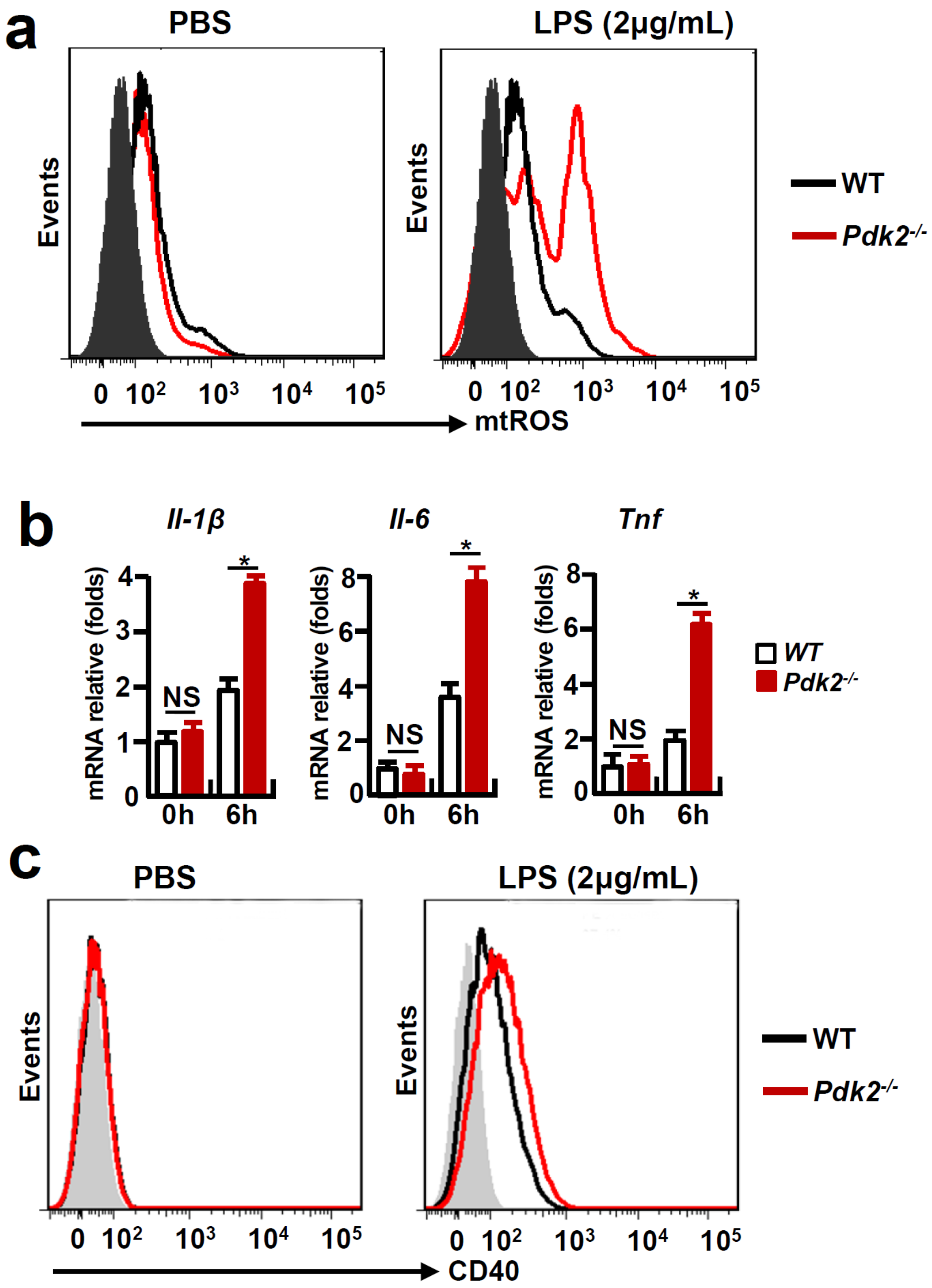

Supplementary Figure 8 *Pdk2* deficiency promotes macrophage activation after LPS exposure *in vitro*. M-CSF-induced BMDMs were administrated with LPS (2 μg/ml) for 6h, and (**a**) mtROS secretion was detected by flow cytometry. (**b**) The mRNA level of macrophage M1-like genes, including *Il-1β*, *Il-6* and *Tnf*, was compared by qRT-PCR, n=5 *per* group. (**c**) Surface expression of CD40 was detected by flow cytometry. Error bar, s.e.m. **p*<0.05 and NS, *p*>0.05 (Student's *t*-test), Data are representative of three independent experiments.

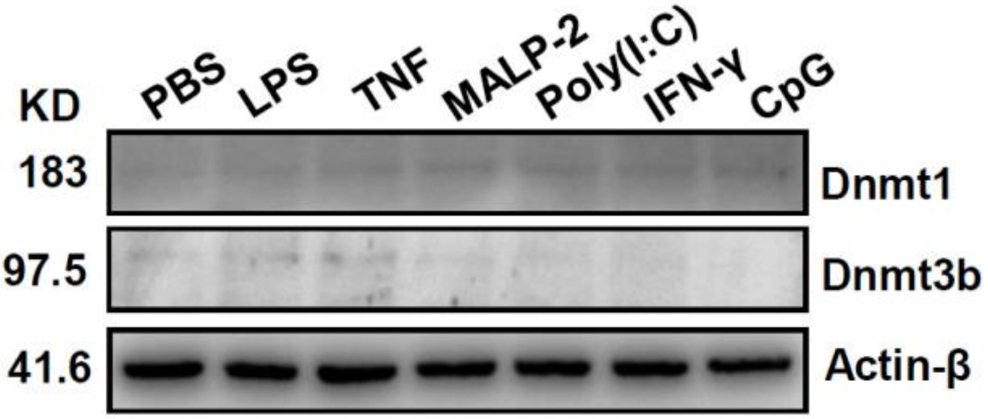

Supplementary Figure 9 The expression of Dnmt1 and Dnmt3b in macrophages was not affected by proinflammatory stimuli. M-CSF-induced BMDMs were treated with proinflammatory mediators for 12h, and the expression of Dnmt1 and Dnmt3b was evaluated by Western-blot. Data are representative of three independent experiments.

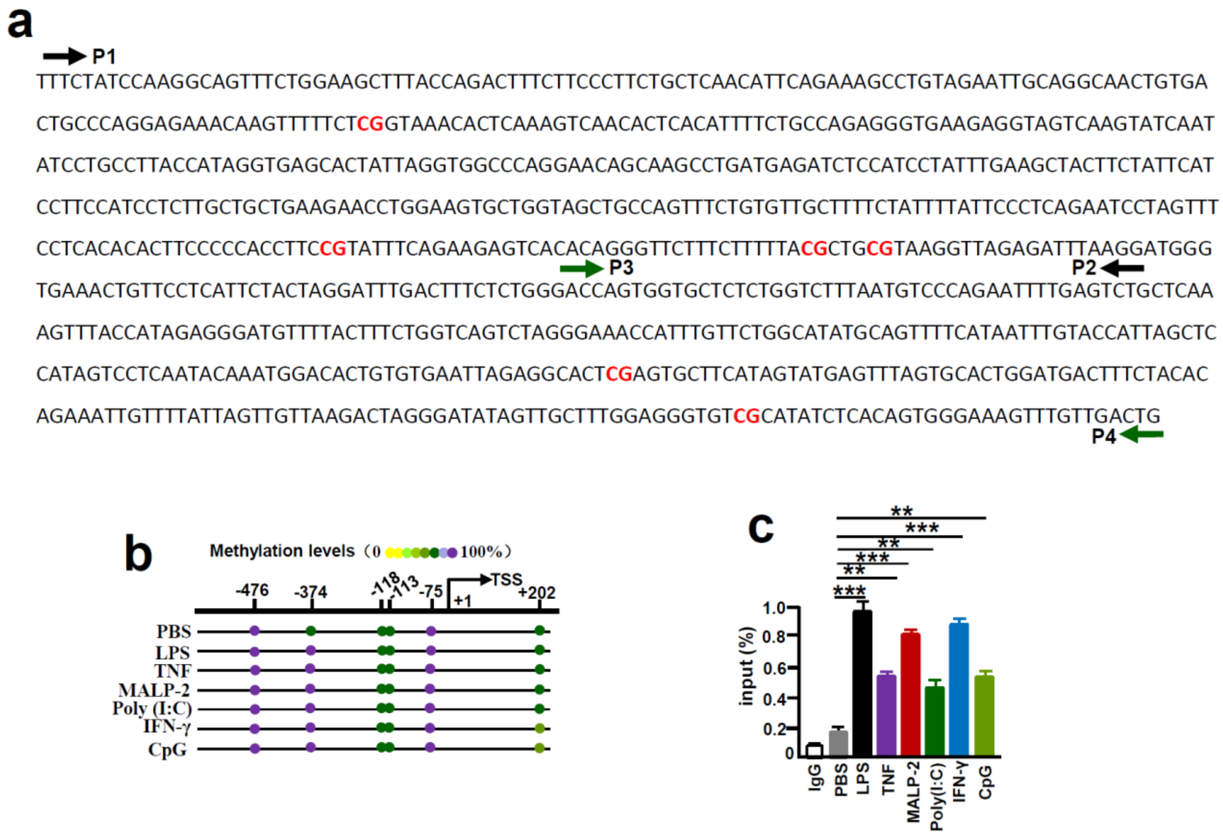

Supplementary Figure 10 *Vsig4* gene promoter is methylated by proinflammatory stimuli in macrophages. (a) A 825bp of cDNA fragments from proximal putative promoter of mouse *Vsig4* gene were amplified to analyze the quantitative methylation by Sequenom MassARRAY platform. The fragments were divided into two regions (promoter region-1 and promoter region-2), and the methylation levels in promoter region-1 was detected by P1 and P2, on the other hand, the methylation levels in promoter region-2 was detected by P3 and P4. The CpG islands are indicated by red characters. **(b)** BMDMs were treated with proinflammatory mediators for 12h, and the methylation levels of CpG islands are shown. The colors of each circle represent the methylation level of each corresponding CpG unit. Results of quantitative methylation analysis r are shown in a color scale: yellow (~0% methylation), green (~50% methylation), and dark blue (~100% methylation). TSS, transcriptional start site. **(c)** ChIP-qPCR assay showing the enrichment of Dnmt3a binding to -374bp of *Vsig4* promoter region in BMDMs after stimulated with proinflammatory factors. Error bar, s.e.m. **p<0.01 and ***p<0.001 (Student's t-test).

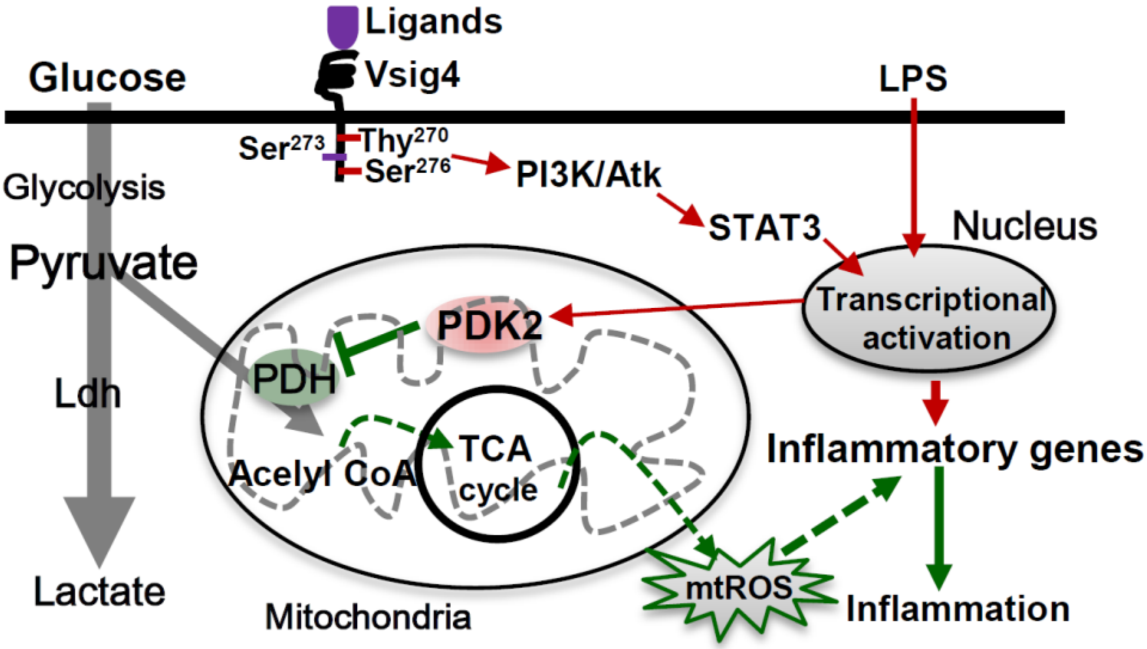

Supplementary Figure 11 VSIG4 reprograms mitochondrial metabolism to attenuate M1 macrophage activation. VSIG4 signalling triggers PI3k/Akt/Stat3 cascades, which promotes metabolic gene *Pdk2* expression and subsequently attenuates PDH activity, leading to limitation of OXPHOS and consequent mtROS secretion, which in turn inhibits the expression of M1 genes.

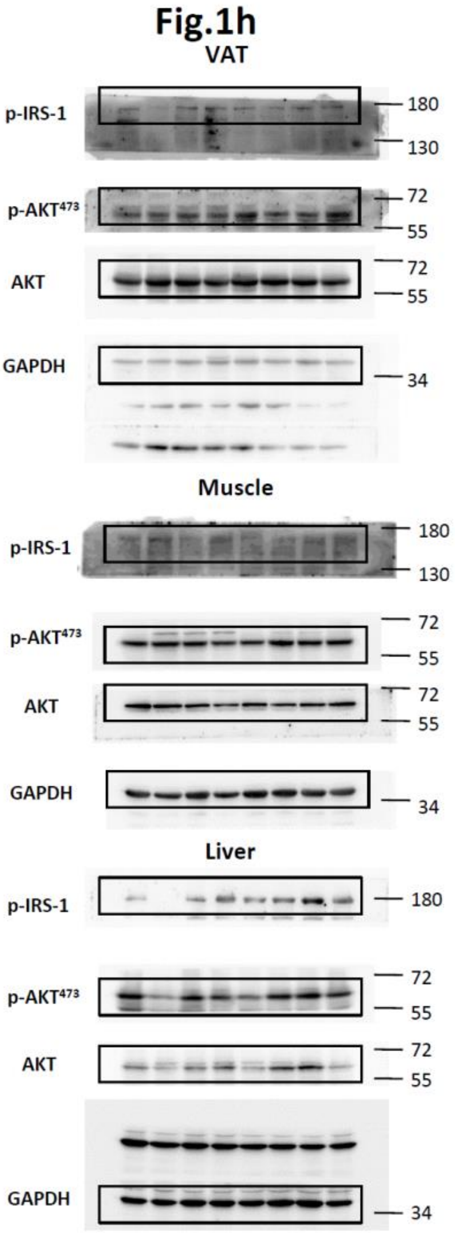
Fig.1h
VAT
p-IRS-1
180
130
p-AKT473
72
55
AKT
72
55
GAPDH
34
Muscle
p-IRS-1
180
130
p-AKT473
72
55
AKT
72
55
GAPDH
34
Liver
p-IRS-1
180
p-AKT473
72
55
AKT
72
55
GAPDH
34

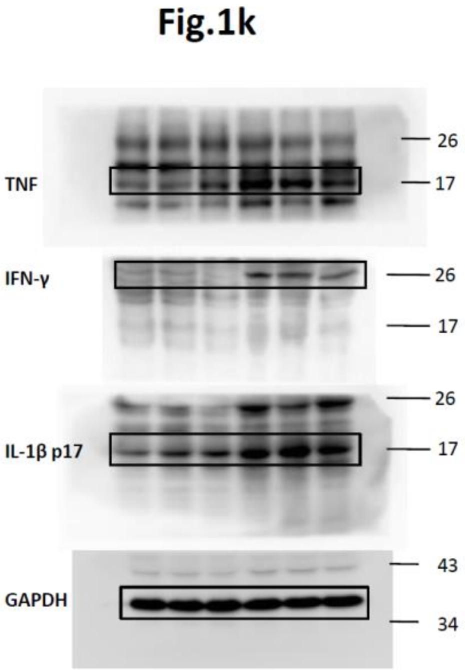
Fig.1k
TNF
26
17
IFN-γ
26
17
IL-1β p17
26
17
GAPDH
43
34

Fig.2g

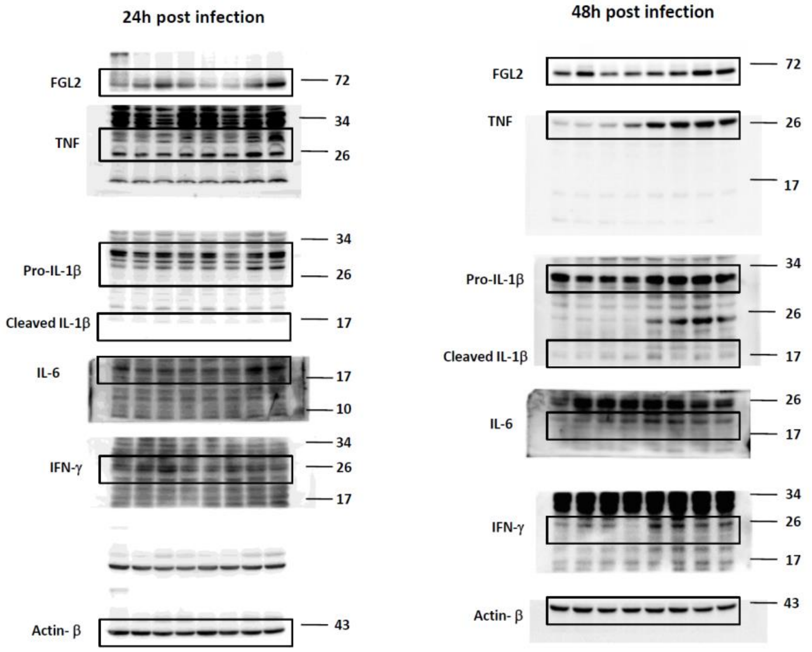
24h post infection
FGL2
72
TNF
34
26
Pro-IL-1β
34
26
Cleaved IL-1β
17
IL-6
17
10
IFN-γ
34
26
17
Actin- β
43
48h post infection
FGL2
72
TNF
26
17
Pro-IL-1β
34
26
Cleaved IL-1β
17
IL-6
26
17
IFN-γ
34
26
17
Actin- β
43

Fig. 3c

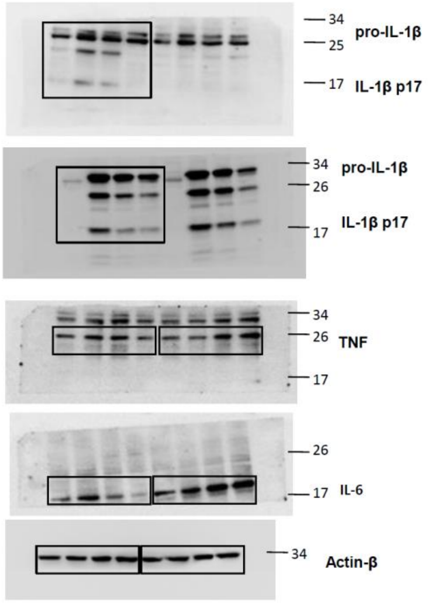
34
25
17
pro-IL-1β
IL-1β p17
34
26
17
pro-IL-1β
IL-1β p17
34
26
17
TNF
26
17
IL-6
34
Actin-β

Fig.5b

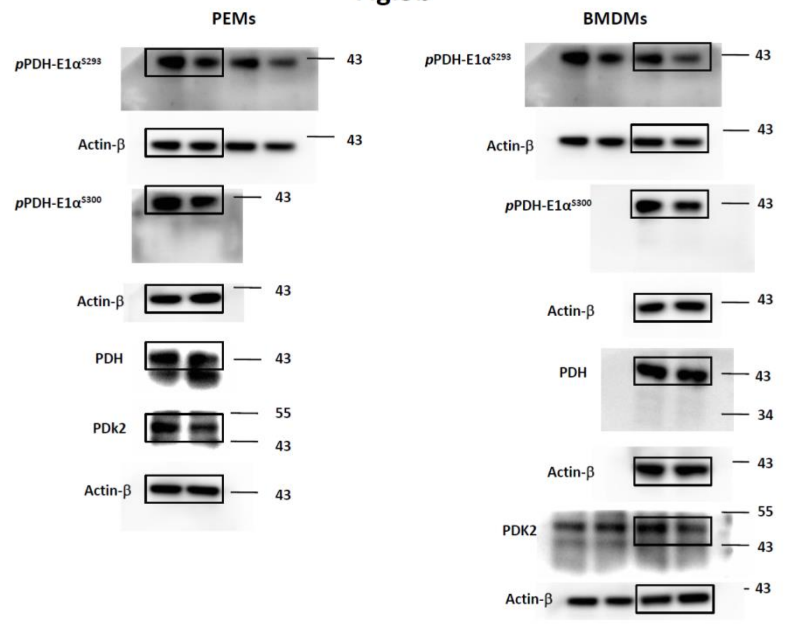

Fig.5e

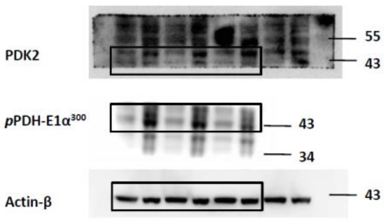

Fig.5d

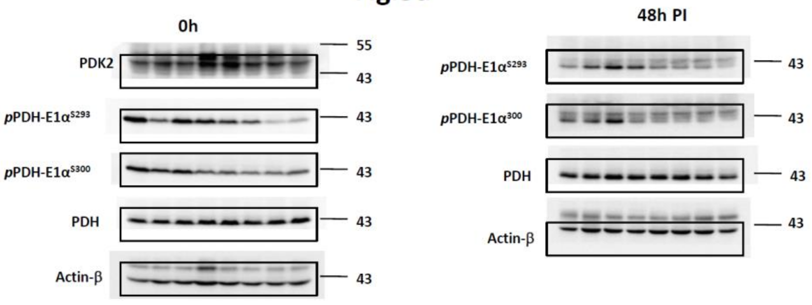

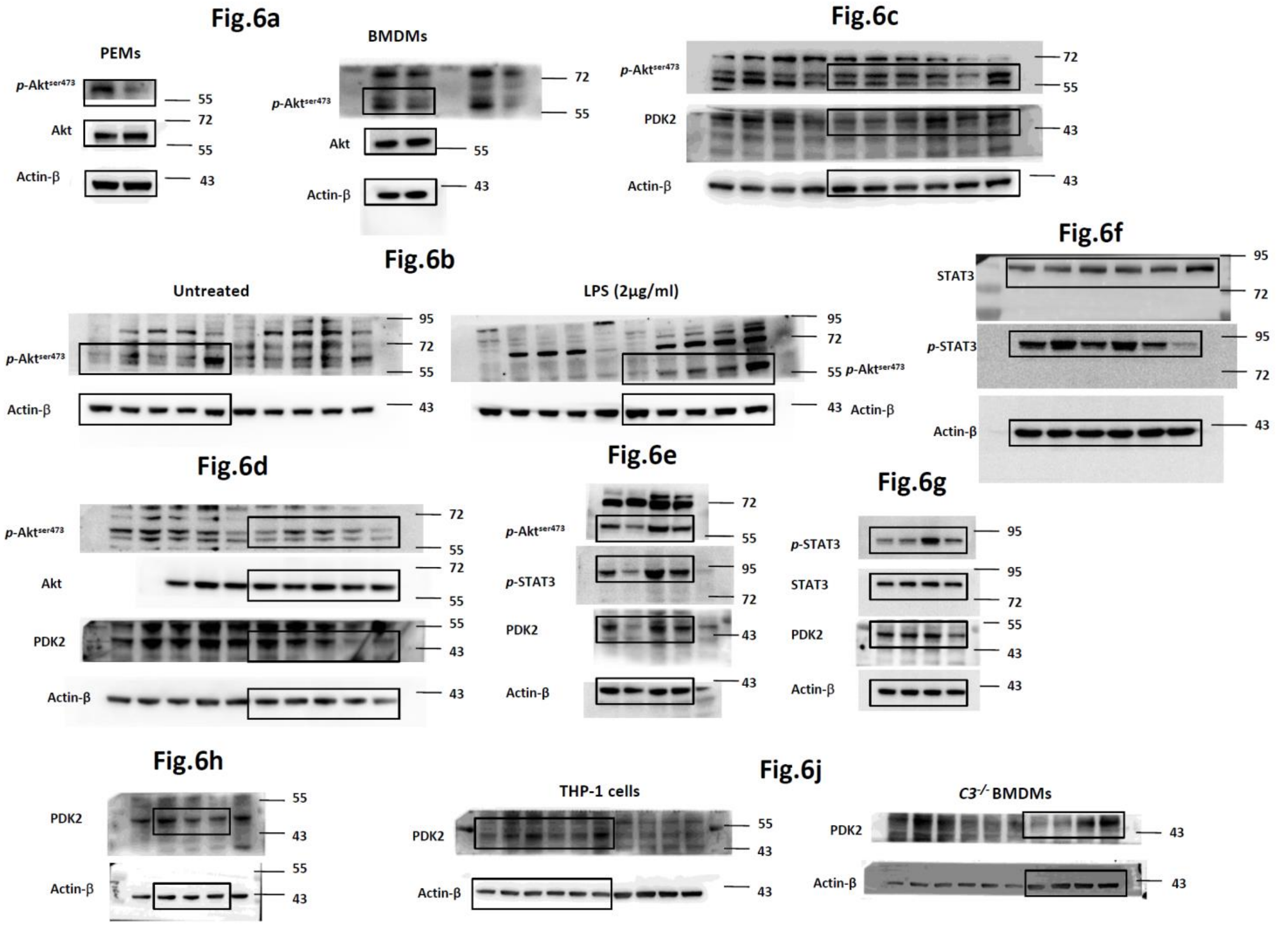

Fig.6a
PEMs
p-Akt ser473
Akt
Actin-β
55
72
55
43
BMDMs
p-Akt ser473
Akt
Actin-β
72
55
55
43
Fig.6c
p-Akt ser473
PDK2
Actin-β
72
55
43
43
Fig.6b
Untreated
LPS (2μg/ml)
p-Akt ser473
Actin-β
95
72
55
43
95
72
55 p-Akt ser473
43 Actin-β
Fig.6f
STAT3
p-STAT3
Actin-β
95
72
95
72
43
Fig.6d
p-Akt ser473
Akt
PDK2
Actin-β
72
55
72
55
55
43
43
Fig.6e
p-Akt ser473
p-STAT3
PDK2
Actin-β
72
55
95
72
43
43
Fig.6g
p-STAT3
STAT3
PDK2
Actin-β
95
95
72
55
43
43
Fig.6h
PDK2
Actin-β
55
43
55
43
Fig.6j
THP-1 cells
PDK2
Actin-β
55
43
43
C3-/- BMDMs
PDK2
Actin-β
43
43

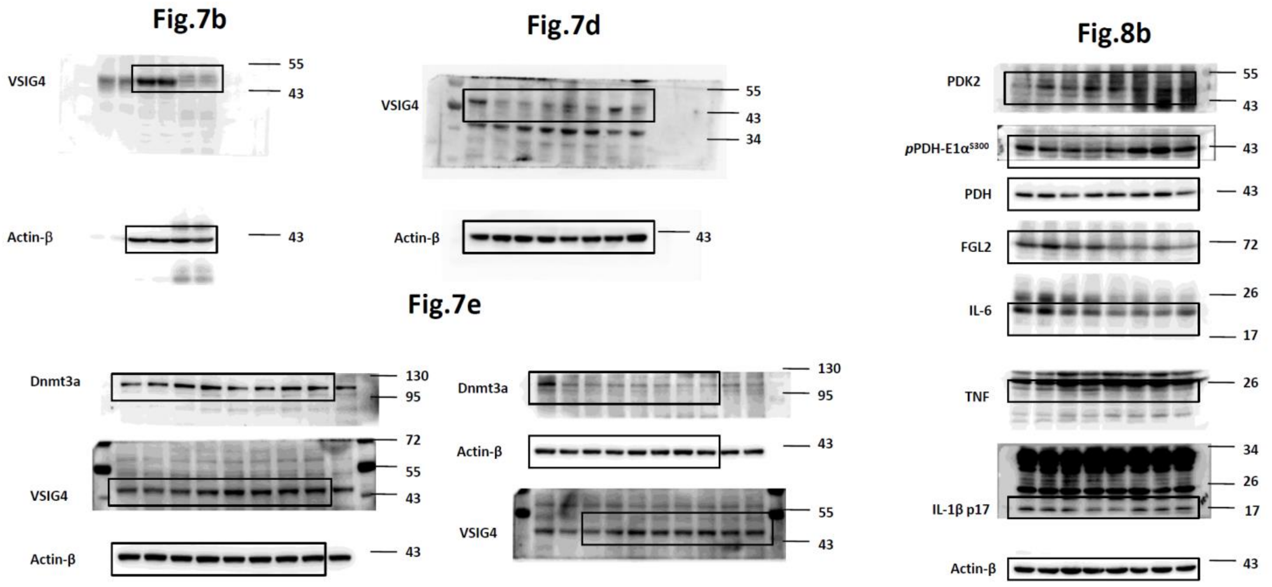

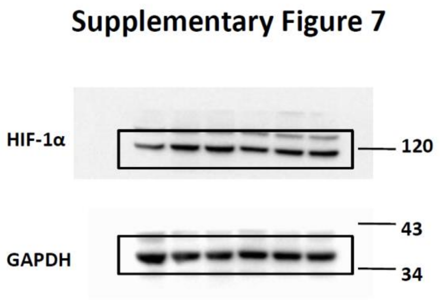

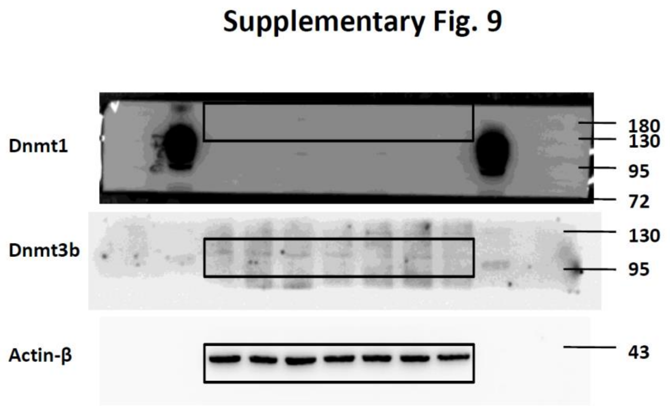

Supplementary Figure 12 Uncropped western blot images.

Supplementary table 1 The quantitation of all CpG's around the *Vsig4* promoter region was detected by Sequenom MassARRAY platform.

Position of CpG (relative to TSS)	PBS	LPS	TNF	MALP-2	Poly(I:C)	IFN-γ	CpG
+202 bp	0.89	0.88	0.74	0.88	0.88	0.89	0.74
-75 bp	0.98	1.00	0.95	1.00	1.00	1.00	0.93
-113bp and -118bp	0.79	0.85	0.76	0.64	0.62	0.67	0.72
-374 bp	0.75	1.00*	1.00*	1.00*	1.00*	0.98*	0.94*
-476 bp	1.00	0.97	0.94	0.98	0.96	0.97	0.94

*Over 20% increase in DNA methylation than PBS- treated group was considered significant different. **TSS:** Transcription Start Site.

Supplementary Table 2 Primers for qRT-PCR.

gene	Sense	Anti-sense
Fgl2	5\`-TGGACAACAAAGTGGCAAATCT-3\`	5\`-TGGAACACTTGCCATCCAAA-3\`
Tnf-α	5\`-CACGCTCTTCTGTCTACTGAAC-3\`	5\`-ATCTGAGTGTGAGGGTCTGG-3\`
Ifn-γ	5\`-TCAAGTGGCATAGATGTG GAAG-3\`	5\`-CGCTTATGTTGTTGCTGATGG-3\`
proIL-1β	5' -CAGGCAGGCAGTATCACTCATTG-3\`	5' -CGTCACACACCAGCAGGTTATC-3\`
IL-6	5' -AGCCAGAGTCCTTCAGAGAG-3\`	5' -GATGGTCT TGGTCCTTAGCC-3\`
Pdk1	5'-CAGCACTCCTTATTGTTCGGTG-3	5' -TGTCTGTCCTGGTGATTTCGC-3\`
Pdk2	5'-ACAGGGAGTGCTGGAGTACAAGG-3\`	5'-TGGAGATGCGGCTGAGGTAGAA-3\`
Pdk3	5'-GCCCAGAAGACCCACGAGTT-3\`	5'-TACTGCTAATGAACGGATCAAACCC-3\`
Pdk4	5'-ACCCAAACTGTGATGTGGTAGCA-3\`	5'-ATGTGGTGAAGGTGTGAAGGAA-3\`
Vsig4	5'-GGATCCCACCCCACCCTAAAAACA-3\`	5'-CTCGAGTCAGCAGGCAGGAATAGA-3\`
Atin-β	5'-CACTATCGGCAATGAGCGGTTCC-3\`	5'-CAGCACTGTGTTGGCATAGAGGTC-3\`

Supplementary Table 3 Primers for whole gene expression of *Pdk2*, *Vsig4*, *Vsig4* truncation mutants and *Vsig4* site-directed mutagenesis.

Gene	Sense	Anti-sense
Pdk2	5'-TAGTCTAGCTAGCATGCGCTGGGTCCGGGC-3'	5'-AGCTAAGCGGCCGCGCTGACCCGATACGTCGATG -3'
Vsig4 $_{\mathbf{whole}}$ (human)	5'-CCGGAATTCATGGGGATCTTACTGGGCCTG-3'	5'-AAGGAAAAAAGCGGCCGCACAGACACTTTTGCCCTCAGT-3'
Vsig4 $_{\mathbf{whole}}$ (mouse)	5'-AGTCGCTAGCCACCATGGACTACAAGGACGACGATGACAAGCTAATAGTGCTCACCTATGG-3'	5'-AGTCGGATCCCTCTTGTTGGAATGTCCTGCA- 3'.
Vsig4 $_{\mathbf{\Delta 119\text{-}280}}$	5'-TAGTCTAGCTAGCATGCGCTGGGTCCGGGC-3'	5'-AGTCGGATCCCTGGCTGAGGCAAGGATCATCA- 3'
Vsig4 $_{\mathbf{\Delta 257\text{-}280}}$	5'-TAGTCTAGCTAGCATGCGCTGGGTCCGGGC-3'	5'-AGTCGGATCCTGGTTCATCAGTTGCGATGGTA-3'
Vsig4 $_{\mathbf{\Delta 267\text{-}280}}$	5'-TAGTCTAGCTAGCATGCGCTGGGTC	5'-AGCTAAGCGGCCGCGCTGACCCGAT

	CGGGC-3'	ACGTCGATG-3'
Vsig4 $Thr^{270}\rightarrow Ala^{270}$	5'-CCAGGAGTACCAAATAGCCATCAGATCAACAAT-3'	5'-ATTGTTGATCTGATGGCTATTTGGTACTCCTGG-3'
Vsig4 $Ser^{273}\rightarrow Ala^{273}$	5'-CCAAATAACCATCAGAGCAACAATGTCTATTCC-3'	5'-GGAATAGACATTGTTGCTCTGATGGTTATTTGG-3'
Vsig4 $Thr^{274}\rightarrow Ala^{274}$	5'-AATAACCATCAGATCAGCAATGTCTATTCCTGC-3'	5'-GCAGGAATAGACATTGCTGATTGATGGTTATT-3'
Vsig4 $Ser^{276}\rightarrow Ala^{276}$	5'-ATCAGATCAACAATGGCTATTCCTGCCTGC-3'	5'-GCAGGCAGGAATAGCCATTGTTGATCTGAT-3'

Supplementary Table 4 Primers for shRNA.

Gene	Sense	Anti-sense
Pdk2	5'-AATTGAGAAGACGTCATTCACTTTCCTCGAGGAAAGTGAATGACGTCTCTTTTTTTAT-3'	5'-AAAAAAAGAGAAGACGTCATTCACTTTCCTCGAGGAAAGTGAATGACGTCTTCTC-3'.
Stat3 #1	5'-CCGGCAGCACAACCTTCGAATCCTCGAGGATTCTTCGAAGGTTGTGCTGTTTTTG-3'	5'-AATTCAAAAACAGCACAACCTTCGAAGAATCCTCGAGGATTCTTCGAAGGTTGTGCTG-3'
Stat3 #2	5'-CCGGCAGCACAACCTTCGAAGAATCCTCGAGGATTCTTCGAAGGTTGTGCTGTTTTG-3'	5'-AATTCAAAAACAGCACAACCTTCGAAGAATCCTCGAGGATTCTTCGAAGGTTGTGCTG -3'

Supplementary Table 5 Primers of DNMT3a and Pdk2 for CHIP-qPCR.

Gene	Sense	Anti-sense
DNMT3a	5' -CAGAAGAGTCAGACAGGGT-3'	5'-TAAAGACCAGAGAGCACCAC-3'
$Pdk2_{1298}$	5'-TGTTATAACTTGCAGTGTGTG-3'	5'-GTGAGTTTAAGTCCAGCCTG-3'
$Pdk2_{2394}$	5'-CACAGCAAGCCACACATGAG-3'	5'-CGTTTGATGTTAGAATGAAC-3'

Supplementary Table 6 Primers for detecting *VSIG4* promoter methylation.

Gene	Sense	Anti-sense
Vsig4 promoter region-1	5'-TTTTTATTTAAGGTAGTTTTTGGAAG-3'	5'-TAAAAAACAATTTCACCCATCCTTA-3'
Vsig4 promoter region-2	5'-TTTTAGAAGAGTTATATAGGGTTTTTTTT-3'	5'-CAATCAACAAACTTTCCCACTATAAA-3'

Supplementary Table 7 Primers for *Vsig4* promoter construction.

Gene	Sense	Anti-sense
840 bp of *Vsig4* promoter (-840/+1)	5'-gctagcAAGATGGAAAATGCCAGTTGTTAT-3'	5'-GAAGATCTGCCAGTTTCTGTGTTGCTTTTC-3'
